# Ambient mass spectrometry for rapid authentication of milk from Alpine or lowland forage

**DOI:** 10.1038/s41598-022-11178-9

**Published:** 2022-05-05

**Authors:** Alessandra Tata, Andrea Massaro, Giorgia Riuzzi, Ilaria Lanza, Marco Bragolusi, Alessandro Negro, Enrico Novelli, Roberto Piro, Flaviana Gottardo, Severino Segato

**Affiliations:** 1 Experimental Chemistry Laboratory, Istituto Zooprofilattico Sperimentale Delle Venezie, Vicenza, Italy; 2 Department of Animal Medicine, Production and Health, University of Padova, Legnaro (PD), Italy; 3 Department of Comparative Biomedicine and Food Science, University of Padova, Legnaro (PD), Italy

**Keywords:** Biochemistry, Chemistry

## Abstract

Metabolomics approaches, such as direct analysis in real time-high resolution mass spectrometry (DART-HRMS), allow characterising many polar and non-polar compounds useful as authentication biomarkers of dairy chains. By using both a partial least squares discriminant analysis (PLS-DA) and a linear discriminant analysis (LDA), this study aimed to assess the capability of DART-HRMS, coupled with a low-level data fusion, discriminate among milk samples from lowland (silages vs. hay) and Alpine (grazing; APS) systems and identify the most informative biomarkers associated with the main dietary forage. As confirmed also by the LDA performed against the test set, DART-HRMS analysis provided an accurate discrimination of Alpine samples; meanwhile, there was a limited capacity to correctly recognise silage- vs. hay-milks. Supervised multivariate statistics followed by metabolomics hierarchical cluster analysis allowed extrapolating the most significant metabolites. Lowland milk was characterised by a pool of energetic compounds, ketoacid derivates, amines and organic acids. Seven informative DART-HRMS molecular features, mainly monoacylglycerols, could strongly explain the metabolomic variation of Alpine grazing milk and contributed to its classification. The misclassification between the two lowland groups confirmed that the intensive dairy systems would be characterised by a small variation in milk composition.

## Introduction

Italian dairy farming relies mainly on high genetic merit cows kept indoor and fed with high energy total mixed rations (TMR) based on maize silage as main fodder. Recently, ensiled forages from grass (Italian ryegrass), cereals (sorghum, wheat) and legume (lucerne) have been introduced into these highly productive cows’ diets to mitigate the environmental negative effects of maize monoculture, as recommended by the common European agriculture policy. As an attempt to improve milk nutritional quality and cheese-making attitude and enhance environmental sustainability, an increasing attention has been given to low input lowland production chains using hays from permanent meadow and lucerne as TMR roughage sources^1^. Meanwhile, in the Alpine farming system, autochthonous lactating cows graze on natural swards with a limited administration of energetic concentrates; a dairy chain producing a highly nutritional milk, mainly processed as protected designation of origin (PDO) cheese, which can be sold at higher prices^2^. Despite the fact that Alpine grazing takes place only during summer season, it contributes to maintain natural resources and preserve botanical biodiversity, which are highly valuable ecosystem services improving environmental sustainability.

Even though there are many factors that can alter milk quality, such as breed, season, number and stage of lactation^3,4^, feed management is certainly one of those with the strongest impact^5–7^, especially if Alpine production is compared with lowland systems. Although many studies have already investigated the effects of maize silage-, hay- or alpine-feeding strategies on milk metabolomics profile^4,8^, understanding the relationship between feeding system and the wide pool of biomarkers useful to authenticate the milk dairy chain is still a challenge. Moreover, a comprehensive metabolomics approach allows to investigate the biochemical pathways involved in the synthesis of milk constituents from the rumen to the mammary gland release.

Over the last few years, metabolomics has been implemented and highlighted as a valuable tool to characterise a large number of compounds in milk and other dairy products and determine whether these metabolites are significantly correlated with the feeding system, especially in uncontrolled real farming conditions^9–11^. Such a multi-analytical investigation needs techniques that are powerful and precise but also less time consuming; the ambient ionisation is an example of technique that enables a great simplification and that shortens time required for analysis^12^. Specifically, direct analysis in real time coupled with high resolution mass spectrometry (DART-HRMS) has already been suggested for accurate dairy authentication^13^, prediction of the health status of dairy cows^14^, and toxicants detection in forages^15^. Considering the high quality of mountain milk^16^, determination of the authenticity of milk feeding systems, rapid detection of falsifications and improvements in traceability along dairy production chains can be beneficial. To this aim, the main goal of this research was to assess the capability of DART-HRMS coupled with a low-level data fusion to discriminate milks according to their intensive lowland or extensive Alpine feeding system origins. To verify whether or not this innovative supervised multivariate pattern recognition could be a valid screening technology, a validation was performed on a test set. Furthermore, an investigation on the cow-related metabolic role of the selected DART-HRMS molecular features was performed.

## Materials and methods

### Ethical statement, experimental design and chemical analysis

To carry out on-farm non-invasive researches, no ethical committee oversight is required under Italian legislation. The authors confirm they did not disturb dairy cows in any way, and raw bulk milk sampling was conducted using non-invasive methods in accordance with guidelines approved by the farm veterinarians.

The study involved 20 dairy farms: 14 of them located in the middle of the Italian lowland area called Po Valley (North East of Italy; 45°40’ lat. N, 11°38’ long. E) and 6 of them in the Alpine area of South-Tyrol (46°33’ lat. N, 11°34’ long. E). The selection of the farms was aimed at representing the average herd size, breeds and milk production characterising both the intensive (lowland) and extensive (Alpine) dairy production chains^12^. On the lowland farms, the lactating dairy cows were fed with TMR, meanwhile, on the Alpine farms, they grazed on natural pastures and received an energetic concentrate supplement daily. All forages were produced on the farms although some concentrate feeds were purchased. According to the main roughage source, three diets were formulated: (i) a mix of maize/grass and legume silages (MMS); (ii) permanent meadow and lucerne hays (HAY); (iii) Alpine pasture (APS). The main ingredients and the proximate composition of the dietary groups are summarised in Table 1. Over a 1-year experimental period, 70 raw bulk milk samples were collected from the lowland farms (MMS = 38 and HAY = 32) across the four seasons, and 18 samples from the Alpine farms (APS = 18) during summer; thus, a total of 88 samples were analysed for milk quality traits and DART-HRMS signatures.

**Table 1:** Diet formulation and proximate composition (average ± standard deviation) of the feeding groups based on the main forage source (% on dry matter, DM).

	Lowland		Alpine
	Mix maize/crop silages	Meadow/lucerne hays	Alpine pasture
	MMS	HAY	APS
	(*n* = 38)	(*n* = 32)	(*n* = 18)
**Diet ingredients (% DM)**			
Maize silage	26	-	-
Grass and legume silages	13	9	5
Permanent meadow hay	8	26	4
Lucerne hay	3	10	1
Green grass	2	6	5
Alpine pasture	-	-	60
Energetic concentrates	26	31	12
Protein concentrates	18	13	8
Residual	4	5	5
**Diet composition (% DM)**			
DM, %	55.1 (± 5.0)	62.2 (± 5.2)	51.0 (± 7.2)
Crude protein	14.0 (± 0.5)	13.8 (± 1.0)	14.8 (± 1.5)
Crude fat	2.7 (± 0.4)	2.5 (± 0.5)	2.1 (± 0.4)
Crude ash	7.9 (± 0.7)	7.9 (± 0.6)	8.8 (± 0.6)
aNDF	37.0 (± 1.9)	39.1 (± 3.8)	43.1 (± 4.4)
ADF	21.9 (± 1.4)	22.5 (± 1.9)	25.9 (± 2.2)
Starch	22.4 (± 1.8)	20.3 (± 1.5)	17.1 (± 1.9)

*Energetic concentrates*, maize and barley grain derivates (meal, extruded, rolled, flaked); *protein concentrates*, soybean and sunflower products; *residual*, straw, bran, beet pulps, mineral-vitamin premix; *aNDF*, neutral detergent fibre; *ADF*, acid detergent fibre. For APS, data referred to the grazing period and considered a theoretical daily dry matter intake (kg) per lactating cow of 12, 5 and 3 of pasture, energetic and protein concentrates and a mix of dried and ensiled forages, respectively.

During each milk sampling, lowland TMR, Alpine sward and energetic concentrate supplement samples were collected. As regards the APS group, the ration samples were made by mixing sward and concentrate in a theoretical 60:40 proportion. Ration samples were oven-dried at 60 °C for 48 h and then ground at 0.5 mm with Universal Cutting Mill Pulverisette 19 mill (Fritsch GmbH, Idar-Oberstain, Germany). Subsequently, they were analysed for chemical traits using the AOAC methods for dry matter (DM), crude protein, crude fat, crude ash, and starch, and ANKOM technology for neutral detergent fibre (aNDF) and acid detergent fibre (ADF), as described by De Nardi et al.^17^.

The milk proximate composition (crude protein, casein, fat, lactose) and chemical traits (urea, native pH) were recorded by a Fourier transform mid-infrared (FT-MIR) spectroscopy technique using a MilkoScan FT6000 (Foss Electric A/S, Hillerød, Denmark). Additionally, the somatic cell count (SCC) was performed by a Fossomatic 5000 (Foss Electric A/S, Hillerød, Denmark).

### DART-HRMS analysis

Two different extraction procedures were applied to the milk samples. In the first one, 50 µL of milk were suspended in 1 mL of water and methanol (H_2_O:MeOH; 80:20 v/v) solution (MilliQ water and Methanol HPLC-grade with 99.9% purity, from VWR International, Radnor, USA), vortexed for 30 s, sonicated for 15 min and centrifuged for 5 min at 12,000 x g to extract the polar metabolites^18^. In the second protocol, 50 µL of milk were diluted in 10 mL of pure ethyl acetate (EtAc) (99.9% purity, Carlo Erba Reagents, Cornaredo, Italy), vortexed for 30 s, then sonicated for 15 min to extract the more lipophilic, non-polar metabolites. A volume (1 mL) of extract was pipetted into a small tube and centrifuged for 5 min at 12,000 g. Subsequently, to obtain four datasets, the two methanol:water diluted samples were analysed in negative and positive ion mode respectively; and so were the ethyl acetate diluted samples. This metabolites fractionation allows differentiated analysis and the expansion of the achievable dataset^19^.

The instrumental analysis was carried out by using a DART SVP 100 ion source (IonSense, Saugus, USA) coupled with an Exactive Orbitrap (Thermo Fisher Scientific, Waltham, USA). The DART source was coupled with a Dip-it^(R)^ sampler (IonSense, Saugus, MA, USA). To facilitate the transfer of the ions from the DART source to the mass spectrometer, a VAPUR interface was installed. The distance between the DART gun and the ceramic transfer tube of the VAPUR interface was 12 mm. The parameters of the DART and the Orbitrap analyser were set as described by Riuzzi et al.^19^. The resolution was set to 70,000 FWHM and the mass range was 75–1125 Da in both the positive and negative ion modes. All DART-MS analyses were run with an automated gain control target setting of 3 × 10^6^. Melting point tubes were inserted into the autosampler holder, and then 5 μL of each extract were spotted individually onto them. Subsequently, the spotted melting point tubes were automatically moved at a constant speed of 0.3 mm/s through the DART gun exit and ceramic tube of the VAPUR interface. The time of desorption from the surface of each tip was about 20 s.

The samples were analysed in triplicate, and XCalibur QualBrowser software (Thermo Fisher Scientific, Waltham, USA) was used to visualise the entire mass spectra in a .raw format. These mass spectra were converted to mzML files by using Proteowizard^20^ and then opened with mMass software (http://www.mmass.org/) to interpret the mass spectrometry data. The *m/z* values were tentatively assigned by consulting the online libraries METLIN (https://metlin.scripps.edu) and HUMAN METABOLOME DATABASE (www.hmdb.ca). Prior to statistical analysis, the mass spectra of the four datasets (two extraction solvents and two ion modes) were converted into .csv files with the Rstudio 3.6.1 software (RStudio Team, 2016; RStudio Integrated Development for R; RStudio, Inc., Boston, USA).

### Statistical analysis

Milk proximate composition and chemical traits were analysed by using a linear mixed model that included the fixed effects of the dietary feeding group and the random effect of the farm (SAS PROC MIXED). Pairwise comparisons among levels of all the factors were performed by using Bonferroni correction. The hypotheses of the linear model on the residuals were graphically assessed. This first statistical model was performed by using SAS 9.4 software (SAS Institute Inc., Cary, NC, USA).

**Figure 1:**
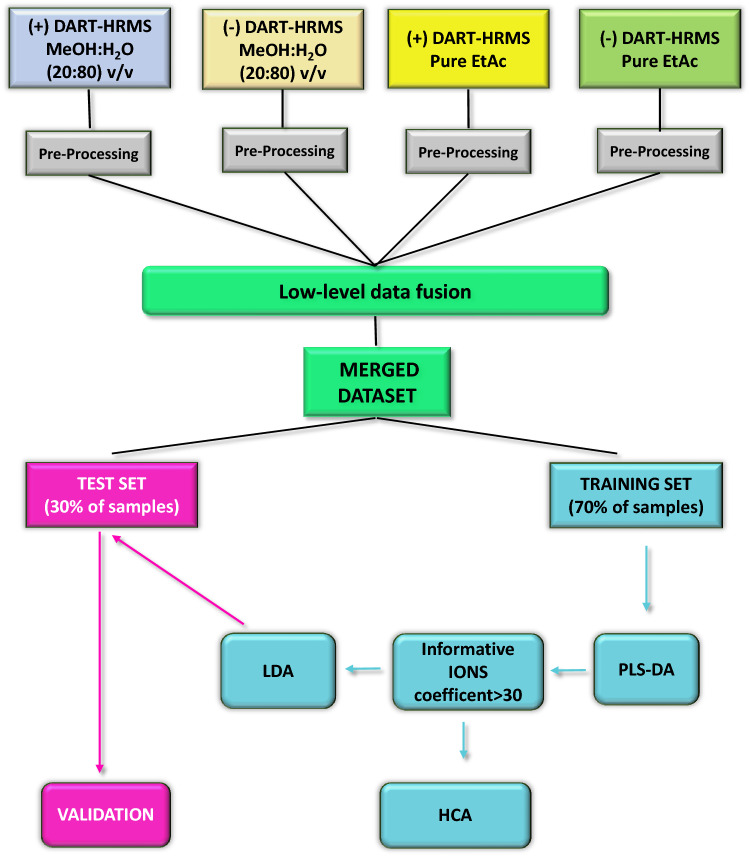
Flow chart of the experimental design and statistical analysis of the (+ / −) DART-HRMS metabolites. After DART-HRMS data pre-processing (TIC normalisation, signal alignment and signal filtering), the four datasets (two dilutions per two ion modes) were submitted to low-level data fusion and the merged dataset was randomly separated into a training (n = 63) and a test (n = 25) set. A partial least squares discriminant analysis (PLS-DA) was performed on the training set and the outcomes were plotted in a scatter gram (see Fig. 2). A hierarchical cluster analysis (HCA) was performed on the 25 selected ions (coefficient > 30) by Pearson distance criterion and generating a heat map (see Fig. 3). The 25 selected ions were used to build a linear discriminant analysis (LDA) model that was validated on the test set (see Table 4).

The triplicate mass spectral data were averaged and statistically analysed by using the Rstudio 3.6.1 software and the MetaboAnalyst 5.0 web portal (www.metaboanalyst.ca) for comprehensive and integrative metabolomic data analysis^21^. The isotopes were removed from the signals recorded in the four datasets, and the *m/z* values aligned with a tolerance of 0.008 Da. All ion signals with more than 75% of missing values (no detected ion intensity) were removed. For ions with less than 75% of missing values, those missing values were replaced with half of the value of the lowest recorded *m/z* intensity. The signals of each mass spectrum were also normalised by sum, whereas each feature of all samples was normalised by Pareto scaling. As reported in Fig. 1, the four datasets were merged (concatenated) by using a low-level data fusion approach. The merged dataset was split into a training (70% of the data, *n* = 63) and a test set (30% of the data, *n* = 25). The training set was submitted to a partial least squares discriminant analysis (PLS-DA) with the aim of distinguishing between the three dietary feeding groups. Subsequently, only the ions with coefficients > 30 were retained. The 25 selected ions were submitted to hierarchical cluster analysis (HCA) with Pearson distance and Ward linkage to show the correlation between groups and the selected ions.

The twenty-five informative *m/z* values extrapolated by PLS-DA were used to build a linear discriminant analysis (LDA) model on the training set by using Rstudio 3.6.1. Its capability to correctly classify the samples according to the dietary feeding group was verified on the training set by a tenfold cross validation. Furthermore, the LDA model was performed against the test set withheld previously. As suggested by Segato et al.^22^, the predictions of this verification were arranged in a confusion matrix and a set of statistical measurements was calculated to assess the predictive discriminating capacity of the supervised classifier LDA model based on the 25 most informative ions sorted by the PLS-DA.

## Results

### Milk quality

Milk proximate composition was significantly (*p *< 0.05) affected by the feeding system with APS showing the highest values for crude protein, casein and fat content (Table 2). No differences in milk nutrients were detected between the two lowland dairy chains. Moreover, the feeding system did not affect urea and SCC across feeding groups.

**Table 2:** Effect of the dietary feeding groups on milk proximate composition and quality traits.

	Lowland		Alpine	SEM	*P *value
	MMS	HAY	APS		
**Milk composition (g/100 g)**					
Crude protein	3.46^b^	3.43^b^	3.53^a^	0.03	0.012
Casein	2.66^b^	2.62^b^	2.72^a^	0.03	0.016
Fat	3.98^b^	3.94^b^	4.25^a^	0.09	0.043
Lactose	4.82	4.78	4.82	0.03	0.084
**Milk quality traits**					
SCC score (units)	3.85	4.03	4.07	0.17	0.152
Urea (mg/dL)	24.6	24.3	25.4	1.4	0.420
Native pH	6.66	6.66	6.65	0.01	0.197

*SCC,* somatic cell count as log_2_ (SCC/100,000) + 3; *MMS*, mix maize/crop silages; *HAY*, permanent meadow and lucerne hays; *APS*, Alpine pasture; *SEM*, standard error of the mean. ^a,b^ Least squares means (LSMeans) in a row without a common superscript differ (*p* < 0.05).

### DART-HRMS

DART-HRMS mass spectra are reported in the additional information (Supplemental Figures S1–S4, Additional File 1). The high-resolution mass spectra allowed the detection and subsequent tentative annotation by library search of a variety of small metabolites and lipids including free fatty acids (FFA), monoacylglycerols (MAG), triacylglycerols (TAG), small organic acids and amino acids. As observed in the mass spectra in Figures S1–S4, DART-HRMS revealed differences in the relative abundances of a variety of milk molecular ions related to fatty acids (FA), amino acids and MAG among the three feeding systems. Intra-sample repeatability was assessed by calculating the percentage (%) of relative standard deviation (%RSD), as an indicator of the instrument fluctuations. To this aim, the intensities of the main ions of APS milk mass spectra were monitored during DART-HRMS data acquisition in negative ion mode. The ratio of absolute intensities of the ions of *m/z* 255.2327/281.2484 was chosen to evaluate the intra-sample repeatability. The reproducibility of DART-HRMS data was assessed with independently extracted samples from a different operator and by subjecting them to independent runs. Six mass spectra were collected from HAY and APS milk in negative ion mode at different times. The resulting inter-day %RSD of 11% suggested that the method is characterised by a small degree of variation and thus a high reproducibility^23^.

**Figure 2:**
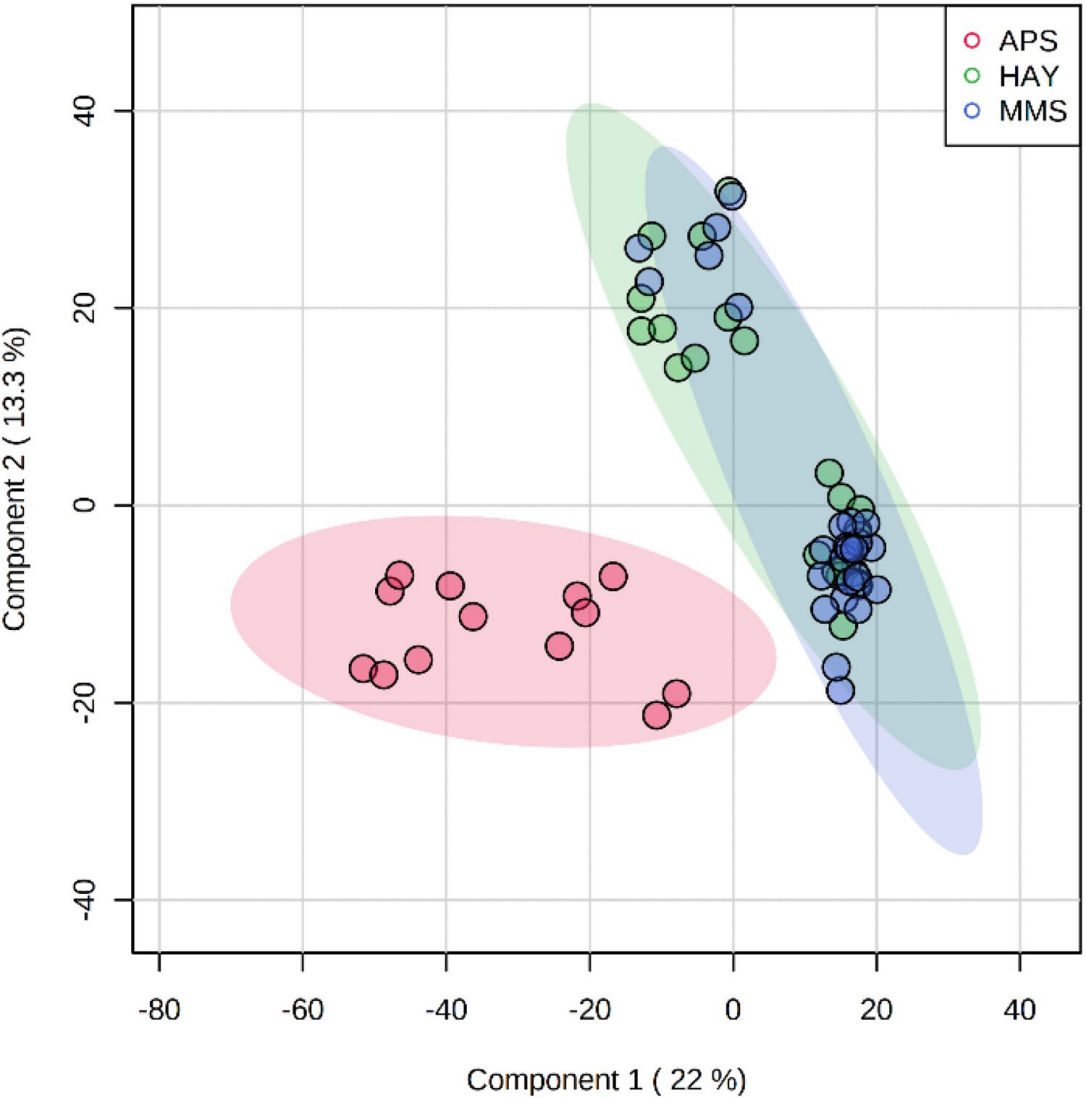
PLS-DA scores plot based on (+ / −) DART-HRMS metabolites on the train set (n = 63). Ninety-five percent ellipses confidence intervals (0.95-CI) are drawn around each centroid of groupings. *MMS*, mix crop/maize silages (blue circles); *HAY*, permanent meadow and lucerne hays (green circles); *APS*, Alpine pasture (red circles).

The outcomes of the PLS-DA performed on the training set extrapolated from the merged dataset showed an accurate separation between the APS milk samples and those from MMS and HAY lowland feeding systems, as a result of the significant differences between their metabolic profiles (Fig. 2). On the contrary, there is no evident separation between MMS and HAY, which overlapped as two spatial subgroups mainly along component 2 of the PLS-DA score plot (Fig. 2). Furthermore, the 0.95-ellipses confidence interval of the APS samples highlighted a wide metabolic variability along the first PLS-DA component.

**Figure 3:**
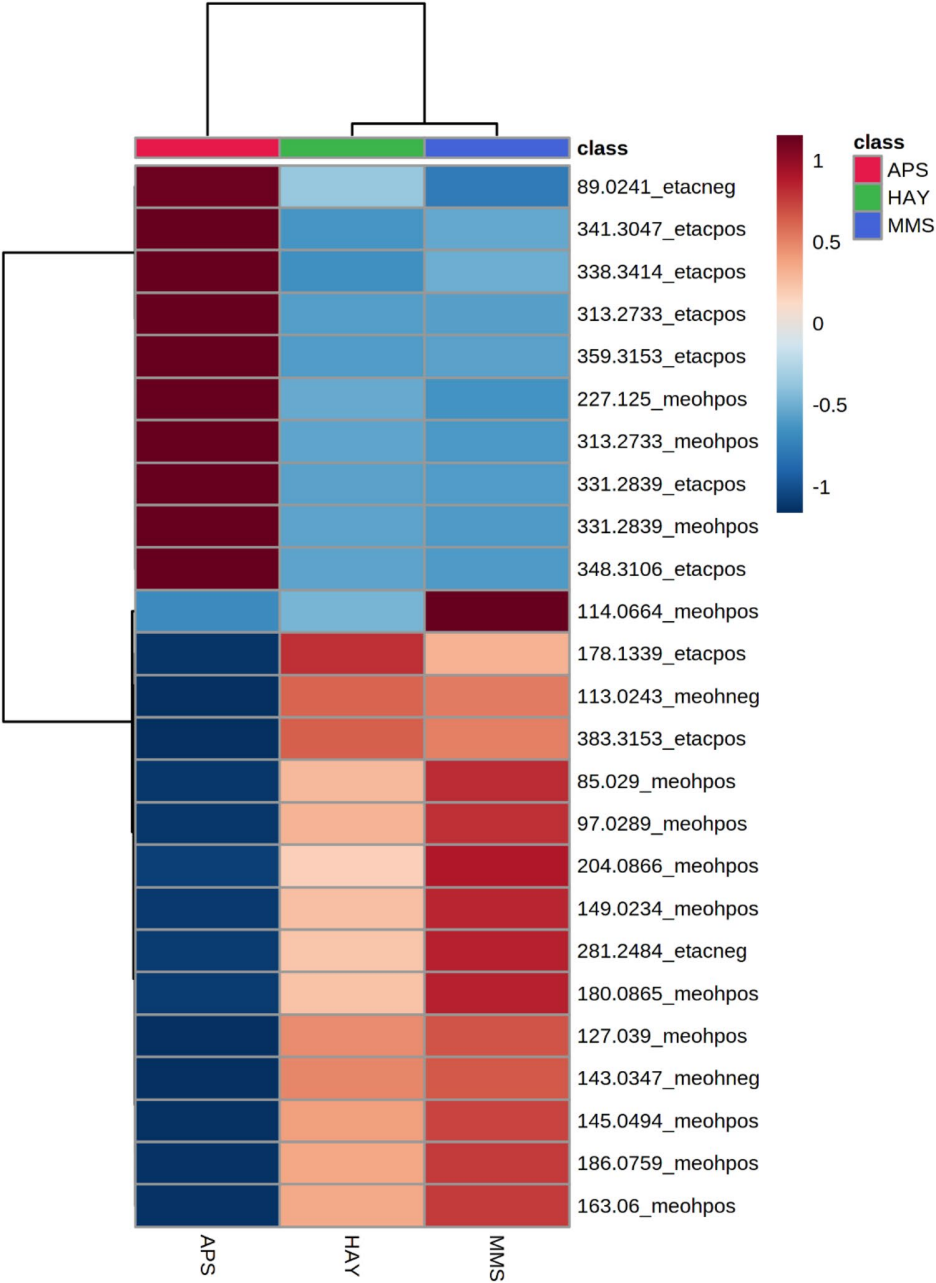
Heatmap obtained by hierarchical clustering analysis (HCA) of the selected milk (+ /−) DART-HRMS ions. The red (positive) and blue (negative) colour scales indicate the degree of correlation between metabolic ions and feeding system; the two shorter Pearson’s distance-tree clusters among the feeding systems (columns) and metabolites (rows) are represented by the branch height (the lower a node is vertical, the more similar its subtree is).

The PLS-DA model allowed extrapolating the 25 most discriminative ions that were submitted to an HCA to estimate the correlation between these DART-HRMS biosignatures and the three feeding systems through a correlation matrix reported in the heat map (Fig. 3). The results of the HCA are presented as a heatmap that shows the correlation between significant ions retrieved by PLS-DA and the three feeding groups. The HCA confirmed the separation into two main sample groups according to the milk production chain based on low (APS) and high-input systems (MMS and HAY).

The tentative assignments of the biomarkers are reported in Table 3. Despite a less evident separation between the two lowland feeding systems, it seems that MMS group is highly correlated (red colour) with *m/z* 114.0664 (protonated creatinine) and mildly correlated with *m/z* 85.029 (ketoacid derivate), 97.0289 (ketoacid derivate), 145.0494 (protonated dimethyl-fumarate), 149.0234 (not assigned), 163.0600 (protonated dehydrated glucose), 180.0865 (ammoniated dehydrated glucosamine), 186.0759 (not assigned), 204.0866 (protonated dehydrated N-acetyl-glucosamine), 281.8424 (deprotonated oleic acid). The HAY group was highly correlated with the ion of *m/z* 178.1339 (ammoniated norgramine) and mildly correlated with *m/z* 113.0243 (deprotonated dehydrated acetolactate) and *m/z* 383.3153 (MAG 20:2), even if these last two *m/z* values seemed to be correlated also with the MMS milk. All of these ions associated with the lowland production chains are strongly negatively correlated (blue colour) with APS. The APS milk showed a high positive correlation with the deprotonated ions of *m/z* 89.0241 (deprotonated lactic acid), the protonated ions of *m/z* 313.2733 (MAG 16:0) and 359.3153 (MAG 18:0) and their dehydrated forms of *m/z* 331.2839 and 341.3047. The APS based milk is also characterised by a high relative intensity of the non-assigned ion of *m/z* 227.125 and the ammoniated MAG (16:0) ion of *m/z* 348.3106.

**Table 3:** Discriminative (+ /−) DART-HRMS metabolites detected in milk samples according to the dietary feeding groups.

Feeding group	DART-HRMS *m/z*	Theoretical *m/z*	Error (ppm)	Elemental formula	Type of ion	Instrument ion mode and extraction solvent	Tentative assignment
MMS	114.0664	114.0662	1.75	C_4_H_7_N_3_O	[M + H]^+^	( +) MeOH:H_2_O (80:20 v/v)	Creatinine
HAY	113.0243	113.0239	3.54	C_5_H_8_O_4_	[M−H−H_2_O]^−^	(−) MeOH:H_2_O (80:20 v/v)	Acetolactate
	178.1339	178.1339	0	C_10_H_12_N_2_	[M + NH_4_]^+^	( +) Pure EtAc	Norgramine
	383.3153	383.3156	− 0.78	C_23_H_42_O_4_	[M + H]^+^	( +) Pure EtAc	MAG (20:2)
MMS/HAY	85.0290	85.0290	0	C_4_H_6_O_3_	[M−H_2_O + H]^+^	( +) MeOH:H_2_O (80:20 v/v)	Ketoacid derivate
	97.0289	97.0290	1.05	C_5_H_6_O_3_	[M−H_2_O + H]^+^	( +) MeOH:H_2_O (80:20 v/v)	Ketoacid derivate
	127.0390	127.0390	0	C_6_H_6_O_3_	[M + H]^+^	( +) MeOH:H_2_O (80:20 v/v)	Methyl 2-furoate
	143.0347	143.0344	2.1	C_30_H_48_O_3_	[M−H]^−^	(−) MeOH:H_2_O (80:20 v/v)	3-hydroxy-2-methylglutarate 2-hydroxy-2-ethylsuccinate
	145.0494	145.0495	0.7	C_6_H_8_O_4_	[M + H]^+^	( +) MeOH:H_2_O (80:20 v/v)	Dimethyl fumarate
	149.0234	–		–	–	( +) MeOH:H_2_O (80:20 v/v)	–
	163.0600	163.0607	− 4.3	C_6_H_12_O_6_	[M−H_2_O + H]^+^	( +) MeOH:H_2_O (80:20 v/v)	Glucose
	180.0865	180.0861	2.22	C_6_H_12_O_6_	[M + NH_4_−H_2_O]^+^	( +) MeOH:H_2_O (80:20 v/v)	Glucosamine
	204.0866	204.0872	− 2.9	C_8_H_15_NO_6_	[M−H2O + H]^+^	( +) MeOH:H_2_O (80:20 v/v)	N-acetyl-glucosamine
	281.2484	281.2486	− 0.7	C_18_H_34_O_2_	[M−H]^-^	(−) Pure EtAc	Oleic acid
APS	89.0241	89.0244	− 3.37	C_3_H_6_O_3_	[M−H]^−^	(−) Pure EtAc	Lactic acid
	227.1250	–		–	–	( +) MeOH:H_2_O (80:20 v/v)	–
	313.2733	313.2743	− 3.19	C_19_H_38_O_4_	[M−H_2_O + H]^+^	( +) Pure EtAc ( +) MeOH:H_2_O (80:20 v/v)	MAG (16:0)
	331.2839	331.2843	− 1.2	C_19_H_38_O_4_	[M−H]^+^	( +) Pure EtAc ( +) MeOH:H_2_O (80:20 v/v)	MAG (16:0)
	341.3047	341.3056	− 2.05	C_21_H_42_O_4_	[M−H_2_O + H]^+^	( +) Pure EtAc	MAG (18:0)
	348.3106	348.3108	− 0.57	C_19_H_38_O_4_	[M + NH_4_]^+^	( +) Pure EtAc	MAG (16:0)
	359.3153	395.0958	− 1.39	C_21_H_42_O_4_	[M−H]^+^	( +) Pure EtAc	MAG (18:0)

*MMS*, mix maize/crop silages; *HAY*, permanent meadow and lucerne hays; *APS*, Alpine pasture; *MAG*, monoacylglycerol.

The discriminative capacity of the LDA classification model, based on the 25 selected ions and carried out on the test set, was reported in a confusion matrix (Table 4). This validation highlighted a reliable and accurate capacity of the classification model to recognise the APS samples as indicated by a set of predictive statistics equal to 1.00. Moreover, the lowland samples were misclassified between each other, and never with the APS ones. The HAY samples seemed to be recognised better than the MMS ones, even if the accuracy and Matthews correlation coefficient (MCC) values indicate a moderate discriminative capacity of the PLS-DA discriminant algorithm.

**Table 4:** Confusion matrix of the linear discriminant analysis (LDA) based on (+ / −) DART-HRMS metabolites; the validation was carried out on the test set (*n* = 25).

Predicted	Actual		
	MMS	HAY	APS
MMS	3	3	0
HAY	8	6	0
APS	0	0	5
Total	11	9	5
Sensitivity	0.27	0.67	1.00
Specificity	0.79	0.50	1.00
Accuracy	0.56	0.56	1.00
Precision	0.50	0.43	1.00
Matthews correlation coefficient	0.07	0.16	1.00

*MMS*, mix maize/crop silages; *HAY*, permanent meadow and lucerne hays; *APS*, alpine pasture.

## Discussion

The significant highest concentration of milk constituents observed in APS-samples could be explained as a strategy of Alpine dairy farmers to enhance milk quality and cheese making attitude by both rearing medium genetic merit lactating cows and limiting their daily milk yield^24^. However, a clear explanation for the response of milk nutrients to dietary forage seems difficult to achieve because of the different parent forages that were used, which are also harvested at different growing phenological stages. The higher crude protein and casein values recorded for the Alpine grazing cows may be due also to an improvement in ruminal nitrogen retention because of the condensed tannins presence in mountain botanical species^25^. Tannins seemed to partially protect forage protein from ruminal degradation, thus enabling a greater availability of N-protein sources in the mammary gland^26^. As regards to fat content, its significant highest content in APS-samples is likely to be caused by a greater ruminal availability of water-soluble carbohydrates (e.g., sugars and fructans) and other readily fermentable carbohydrates that might have stimulated the synthesis of β-hydroxy-butyric acid, which is positively correlated with milk fat synthesis in the mammary gland^27^. The native plant fatty acids did not affect milk fat content probably because the rumen microbiota biohydrogenation and the further metabolic pathways did not result in any antilipogenic fatty acids, such as rumen intermediate trans C18:1 isomers.

The main goal of this study was to evaluate the capacity of multi-modal DART-HRMS to trace three dairy production chains. The first one is the high-input system based on arable lowland (maize and other cereal silages) and high genetic merit lactating cows; the second one relies on lowland permanent meadow to produce a “hay-milk” that is more suitable for hard cheese making. Finally, the third one is based on a low-input production approach that combines extensive Alpine natural pasture with local low genetic dairy cows and its main purpose is to produce high-value mountain cheeses. Although there are several studies that investigated the effect of these feeding regimens on milk constituents, the majority of them focused on a specific chemical class (lipids, N-compounds, carbohydrate-like substances) and were also carried out under controlled experimental farm conditions^3,28^. On the contrary, through the use of a low-level data fusion, this study simultaneously modelled milk metabolites retrieved from four datasets (polar and non-polar extracts analysed in positive and negative ion modes) obtained in real farming conditions. Indeed, a multimodal mass spectrometric approach was applied as a comprehensive metabolomics fingerprinting to identify informative biomarkers that may be used as production indicators within the labelling process and by policymakers to award dairy farmers. Taking all this into account, DART-HRMS coupled with chemometrics demonstrated its capability to correctly discriminate APS milk samples from the lowland ones confirming that the geographical (e.g., altitude and crop selection) and botanical origin of forage is the driving key factor of mountain milk specificity^9,10^.

Despite low-level data fusion is a strategy that is capable of integrating various data sources and of providing a more comprehensive exploration of the chemical composition, there was still a spatial overlapping between the two lowland feeding clusters in the PLS-DA scatter plot (Fig. 2). Furthermore, the PLS-DA performed on the merged DART-HRMS training set allowed identifying the most discriminative ions. As confirmed by the heatmap (Fig. 3), the major metabolic differences lie between the lowland and the Alpine milks, even though some changes in the relative intensities of the biomarkers can be observed also between MMS and HAY samples. However, the outputs of this research highlighted a spatial arrangement of two indistinguishable lowland subclusters likely due to the variability of the home-grown forages (cropping and harvesting practices) and/or to seasonal effects within the MMS and HAY groups. The influence of these uncontrolled field-related factors on the variability of milk DART-HRMS metabolic profile suggests that the accuracy and reproducibility of newly devised classification algorithms need to be regularly validated by experimental evidences from on-farm investigations.

The model correctly classified all the APS samples with accuracy, sensitivity, specificity and MCC equal to 1.00. The same performance indicators are low for the classification of HAY and MMS due to their high chemical similarity as compared to APS profiles. This indicates that the APS metabolic profiles are very distinct and highly correlated to the grazing nature of milk (Table 4). We can speculate that, in the future, this unique characteristic fingerprint can be exploited for rapid screening of APS milk and detection of production system frauds.

Milk produced by cows on the lowland farms (MMS and HAY) was characterised by a pool of energetic compounds (creatinine, glucose, acetolactate), ketoacid derivates, low-weight molecules (methyl 2-furoate, dimethyl fumarate, norgramine), amines (glucosamine, N-acetyl-glucosamine), and organic acids (3-hydroxy-2-methylglutarate or 2-hydroxy-2-ethylsuccinate and oleic acid). Among these, only creatinine was strongly correlated with the ensiled forages (MMS), meanwhile the HAY feeding group seemed to have a stronger association with acetolactate, norgramine and MAG (20:2). However, many of these metabolites showed correlations with both MMS and HAY feeding systems, thus causing the overlapping highlighted by the PLS-DA (Fig. 2).

Creatinine (*m/z* 114.0664) and its precursor phosphocreatinine were found as potential indicators of stressful feeding conditions for lactating dairy cows, as rapidly mobilizable reserves of energy in skeletal muscles^29,30^. In the MMS group, this stressful conditions may have occurred because of greater daily milk yield performances, even though it was reported also as a biomarker of milk coming from early lactation stages^29^ and of cows’ health status^31^. Despite the low concentration of creatinine and of its derivates in milk, estimating the amount of these amino acid-like energetic compounds can be useful to assess milk nutritional value.

Norgramine (*m/z* 178.1339) seemed to be the DART-HRMS signature that is most correlated with the HAY system. To our knowledge, it was reported as a biomarker of feeding regimes based on a mix of grass and legume^19^, which is similar to the composition of the HAY diet as it is based on dried and ensiled grass and legume forages. As norgramine was found to be strongly negatively (r = -0.85) correlated with APS milk samples, its absence is an indication of Alpine dairy products, although further studies are needed to prove its effectiveness in tracing milk origin. The third ion that was assigned to HAY feeding thesis is monoacylglycerol MAG (20:2) (*m/z* 383.3153). The detection of doubly unsaturated MAG in milk was already reported in literature as MAG fragment ions in a lower mass region but with no clear explanation with regards to their production system and milking and milk conservation methods^13,32^.

With regards to lowland milk samples, two low-weight molecules were detected as methyl 2-furoate (*m/z* 127.0390) and dimethyl fumarate (*m/z* 145.0494) and two isomers of *m/z* 143.0347 were identified as 3-hydroxy-2-methylglutarate or 2-hydroxy-2-ethylsuccinate. Although methyl 2-furoate, a volatile compound, and dimethyl fumarate, a fumarate-derivate, were already detected as compounds of lowland TMR rations mainly based on maize silage and other grass silages (sorghum, wheat, Italian ryegrass), there is not a clear explanation for their correlation with these feeding strategies^13,19,33^. The two isomers (3-hydroxy-2-methylglutarate or 2-hydroxy-2-ethylsuccinate) could be involved in metabolic cycles to support the lactate synthesis in the mammary gland^31^. Regardless of the specific anabolic pathway, these intermediates of gluconeogenesis could be associated with a high-intensity mammary gland activity with their releasing as traces in milk. The synthesis of propionate, one of the main substrates involved in gluconeogenesis, is also supported by pyruvate throughout the succinic pathway, suggesting that the presence of 2-hydroxy-2-ethylsuccinate is proof of a metabolism addressed to support cow’s energy needs for lactation^34^. Moreover, the detection of glucose (*m/z* 163.0600) in the lowland milk samples confirms this hypothesis, as it was already correlated with a high intake of starch and/or rapidly fermentable sources of carbohydrates provided to highly productive dairy cows^31,33^. A higher glucose and lactose concentration in milk was explained as the result of an increased intramammary pressure due to a greater milk production^35^. Oleic acid (*m/z* 281.2484) is both influenced by season and involved in body fat mobilization^36^, and the presence of this FFA may be due to its greater content in milk and derivates from lowland TMR compared with the Alpine grazing systems^9,37^. Finally, so far, N-acetyl-glucosamine has been found as a milk biomarker but its presence was not put into correlation with feeding strategy^33,38^; meanwhile, there are not findings on the presence of ketoacid derivates and glucosamine in milk.

As regards to APS group, the application of DART-HRMS highlighted a wide and specific pool of biosignatures for the authentication of Alpine grazing milk. The metabolic profile of Alpine milk was identified by 11 ions related to lactate and a predominance of MAG molecules characterised by the presence of C16:0 and C18:0. Lactic acid (*m*/*z* 89.0241) could be related to a relatively high SCC^33^, even if we did not find any statistical difference among feeding groups. Conversely to our findings, O’Callaghan et al. reported that L-lactate was more correlated with milk from cows fed a silage-based TMR comparing with those reared outdoors on a lowland perennial pasture^34^. Probably, in the Alpine environment the raw milk microbiota from the entero-mammary and exogenous (teat apex and skin) pathways could play a role in an early chemical alteration (i.e., fermentation) of lactose and oligosaccharides, even if this finding requires further investigation^39^. Traces of milk MAG rich in C18:0 and C16:0 might be also explained through the action of the environmental-specific activity of indigenous lipoprotein lipases (LPL) carried out soon after milking. LPL have an optimum activity at the sn-1 and sn-3 positions of the glycerol backbone, where they are mainly esterified by polyunsaturated FA (PUFA) and short chain FA (SCFA), respectively^40^. Therefore, as a result of the early and sn-1- and sn-3-addressed enzymatic activity of LPL, MAG (C16:0) and (C18:0) remain in milk and, even if in low concentrations, are detected by DART-HRMS. Their identification may be a key-factor in APS milk authentication, even if this outcome needs to be investigated by further lipidomic studies. Large botanical diversity and environmental conditions (e.g., outdoor/pasture grazing vs. indoor/hay) may influence milk microbiota originating from teat skin and the following specific enzymatic activities of the microflora conveyed in milk^29,39^. However, as reported in our previous trial^19^, during DART analysis, TAG undergo in-source fragmentation to yield diacylglycerol and monoacylglycerol fragment ions, as a result of a possible thermal degradation. Whether they derive from in-source DART fragmentations or from hydrolysis phenomena in milk, it is necessary to take into account that lipids are a complex matrix due to the presence of more than 400 FA and more than 1300 of their combinations in 3-acylgricerols (TAG) having specific physical, chemical and nutritional properties^41^. Furthermore, lipid extraction is still a critical issue to be solved as the analytical criterion used to perform their extraction could affect the identification and quantification of specific categories of lipids^32^.

## Conclusions

We demonstrated that DART-HRMS, coupled with low-level data fusion and chemometrics, provides a simplified approach that allows a straightforward and deeper access to chemical composition of milk directly from diluted samples. While the high-input silage-based system is characterised by creatinine, the hay-based milk can be identified by the higher relative intensity of acetolactate, norgramine and MAG (20:2). However, a pool of metabolites is shared by the two lowland systems. The discrimination of Alpine milk was allowed by a predominance of MAG molecules. Therefore, through the precise determination of the chemical profile of the milk and the construction of statistically based classifiers, DART-HRMS could be employed for rapid authentication of Alpine pasture milk from those produced in lowland scenarios. This innovative analytical tool is able to accelerate the alpine pasture-milk authenticity verification and to enhance the traceability systems along milk production chains.

## Supplementary Information

Supplementary Information.
