# Supplementary material for: Ambient mass spectrometry for rapid authentication of milk from Alpine or lowland forage
Source: Sci Rep. 2022 May 5;12:7360. doi: 10.1038/s41598-022-11178-9 (PMC9072378; doi:10.1038/s41598-022-11178-9)
Supplement: Supplementary file 1 — Supplementary Information. [file 41598_2022_11178_MOESM1_ESM.docx]

Figure S1-S4. Ambient mass spectrometry for metabolomic fingerprint of milk according to the feeding system (MMS, mix maize/crop silages; HAY, permanent meadow and lucerne hays; APS, alpine pasture)

Figure S1. DART-HRMS profiles of non-polar extracts of MMS, HAY and APS milks acquired in negative ion mode

Figure S2. DART-HRMS profiles of non-polar extracts of MMS, HAY and APS milks acquired in positive ion mode

Figure S3. DART-HRMS profiles of polar extracts of MMS, HAY and APS milks acquired in negative ion mode

Figure S4. DART-HRMS profiles of non-polar extracts of MMS, HAY and APS milks acquired in positive ion mode
